# Short-Term Memory Deficit Associates with miR-153-3p Upregulation in the Hippocampus of Middle-Aged Mice

**DOI:** 10.1007/s12035-023-03770-5

**Published:** 2023-11-15

**Authors:** Francesca Stabile, G. Torromino, S. Rajendran, G. Del Vecchio, C. Presutti, C. Mannironi, E. De Leonibus, A. Mele, A. Rinaldi

**Affiliations:** 1 Department of Biology and Biotechnologies “Charles Darwin” (BBCD), Sapienza University of Rome, Rome, Italy; 2 Centre for Research in Neurobiology Daniel Bovet (CRiN), Sapienza University of Rome, Rome, Italy; 3 Department of Humanistic Studies, University of Naples Federico II, Naples, Italy; 4 Institute of Molecular Biology and Pathology, c/o Department of Biology and Biotechnology, National Research Council, Sapienza University of Rome, Rome, Italy; 5 Institute of Biochemistry and Cell Biology, National Research Council (IBBC-CNR), Monterotondo (Rome), Italy; 6 Telethon Institute of Genetics and Medicine (TIGEM), Pozzuoli (Naples), Italy

**Keywords:** High-load short-term memory, Ageing, MicroRNAs, miR-153-3p, Hippocampus

## Abstract

The early stages of ageing are a critical time window in which the ability to detect and identify precocious molecular and cognitive markers can make the difference in determining a healthy vs unhealthy course of ageing. Using the 6-different object task (6-DOT), a highly demanding hippocampal-dependent recognition memory task, we classified a population of middle-aged (12-month-old) CD1 male mice in Impaired and Unimpaired based on their short-term memory. This approach led us to identify a different microRNAs expression profile in the hippocampus of Impaired mice compared to Unimpaired ones. Among the dysregulated microRNAs, miR-153-3p was upregulated in the hippocampus of Impaired mice and appeared of high interest for its putative target genes and their possible implication in memory-related synaptic plasticity. We showed that intra-hippocampal injection of the miR-153-3p mimic in adult (3-month-old) mice is sufficient to induce a short-term memory deficit similar to that observed in middle-aged Impaired mice. Overall, these findings unravel a novel role for hippocampal miR-153-3p in modulating short-term memory that could be exploited to prevent early cognitive deficits in ageing.

**Supplementary Information:**

The online version contains supplementary material available at 10.1007/s12035-023-03770-5.

## Introduction

Ageing can be broadly defined as a time-dependent physiological decline that increases the likelihood of incurring in several common diseases (for a review see [1]). The ability to detect early signs of cellular and cognitive senescence and to differentiate between causal factors and those merely associated with unhealthy ageing holds significant potential to prevent or mitigate age-related functional decline from its early stages, with considerable social and economic benefits.

In the long journey of ageing, mild cognitive impairment (MCI) is a transient condition that some individuals experience in middle age before developing dementia (for a review see [2]). People affected by MCI are generally able to lead a normal life, but they begin to show specific impairments in at least one cognitive domain, often including short-term memory (STM) capacity deficits that are usually identifiable only through sophisticated neuropsychological testing [3].

STM capacity refers to the number of items that an individual can remember for a brief period of time (from seconds to minutes). In physiological conditions, STM capacity averages around 7 ± 2 items in humans [4–6], as well as in monkeys and rodents [7, 8]. In a mouse model of MCI, it has recently been shown that subtle STM capacity deficits in middle-aged subjects can predict their overall memory decline at later stages [9]. Although STM is generally associated with the activation of prefrontal regions (see [10, 11], but also [12]), studies in mice [8, 13], monkeys [7, 14] and humans [15] have demonstrated the involvement of the hippocampus (HP) in high-load memory tasks. Middle-aged mice with STM impairment in high-load condition showed defective glutamatergic proteostasis in the HP compared to age-matched cognitively preserved subjects [9]. The dysregulation of proteostasis that occurs at the onset of ageing may be linked to altered expression of microRNAs (miRNAs), which has also been observed in the HP during the ageing process [16–18]. miRNAs are short non-coding RNA of 20–22 bp—highly expressed in the brain—that regulate and fine-tune target mRNAs translation by specifically binding their 3′-untranslated region (3′-UTR) [19].

Several miRNAs have been associated with memory functions and related synaptic plasticity mechanisms [20–25]. For instance, a study from Konopka and colleagues [20] demonstrated that deletion limited to forebrain neurons of the gene Dicer 1—implicated in miRNAs biosynthesis—leads to decreased miRNAs expression and improved performance in the Morris water maze task. Conversely, we have previously demonstrated that overexpression of miR-324-5p in the HP of adult mice interferes with the formation of long-term spatial memory [22]. Alterations in the expression of specific miRNAs in dendrites have been linked to changes in receptors expression and dendritic density [26, 27], suggesting that they can quickly modify gene expression at the synaptic level and may therefore play a crucial role in regulating rapid brain activity, such as that associated with STM. Accordingly, it has been recently shown that sponge-mediated miR-138-5p sequestration in interneurons impairs spatial STM in mice [28]. Although these data suggest that miRNAs fine regulation may be associated with the memory impairments that characterise MCI, such as those in STM capacity, the role of miRNAs in the early phases of ageing remains largely unexplored.

In the present work, we set out to test the hypothesis that miRNAs might be involved in the subtle STM impairment that occurs during early age-related cognitive decline, with the aim of identifying possible miRNA to be used as markers of this stage.

We used a condition of high memory load to reveal early STM deficits in a population of 12-month-old male CD1 mice by testing them in the 6-different object task (6-DOT), which allowed us to distinguish between Unimpaired and Impaired subjects. Microarray screening of the dorsal HP of Impaired vs Unimpaired mice showed a pool of differentially regulated miRNAs between the two groups, including miR-153-3p. Finally, we showed that the miR-153-3p overexpression is functionally involved in the observed STM deficit.

## Material and Methods

### Subjects

All experiments were conducted on naïve outbred CD1 male mice (Charles River, Como). Mice arrived in the animal facility at the age of 5–8 weeks and were housed in groups of 3 to 5 in standard cages (26.8 × 21.5 × 14.1 cm) with enrichment conditions, water and food ad libitum. Experimental rooms were kept under a 12 h light/dark cycle and constant temperature (22 ± 1 °C). Behavioural training was conducted during the light period (from 9:00 to 17:00). At the time of experiments, mice were 3 or 12 months old, and their weight ranged from 30 to 50 g. All the animals were treated in accordance with current Italian and European laws for animal care, and the maximum effort was made to minimise animal suffering.

### Behavioural Procedure

All mice of the 3 and 12 months groups underwent the *6-different object task* (6-DOT [8]). Before starting the procedure, mice were isolated in a waiting cage for 15 min and then subjected to the experimental procedure consisting of three consecutive phases: habituation, study and test. After isolation, the habituation phase began by putting the mouse at the centre of an empty open field (35 × 47 × 60 cm) and letting it free to explore the environment for 10 min. At the end of the habituation phase, mice were put back in the waiting cage for an intertrial interval of 1 min. They were then exposed to the same open field but with six objects, different for shape and material. Mice were left to freely explore the objects until they reached a maximum exploration time of 210 s (35 s *per* object). When they did not reach the maximum exploration criterion, the duration of the study phase was set at 10 min, after which mice were put in the waiting cage for an intertrial interval of 1 min before starting the test phase. During the test phase, mice were exposed to one novel object and five copies of the familiar objects. The test phase lasted 5 min for each mouse. The position of the new object was changed across animals in a random order, while the configuration of objects between study and test phase for a single animal was maintained the same. Exploration was defined as the time in which the nose of the animal was not further than 2 cm from the object [8]. Climbing or staying on the objects was not considered exploration. New object recognition was considered occurring when the new object was explored significantly more than all the familiar objects.

After completion of testing in 6-DOT task, mice were a posteriori segregated in two groups, *Unimpaired* and *Impaired*, based on the amount of time spent exploring the new object and on the mean time spent exploring the familiar ones. The following criterion [9] has been applied: if New > [(Mean Familiar) + (SD Familiar * 1.5), the subject was considered Unimpaired, if New < [(Mean Familiar) + (SD Familiar * 1.5) the subject was considered Impaired.

### RNA Extraction

Immediately after the behavioural task, mice were sacrificed for microarray and reverse transcription-quantitative PCR (RT-qPCR) analysis. Brains were quickly dissected, and dorsal hippocampi were separated according to Mouse Brain Atlas (www.mbl.org). Extracted tissue was immediately transferred into a clean eppendorf tube containing Qiazol reagent (Qiagen, DE) and flash frozen. Samples were stored at − 80 °C until RNA extraction. Total RNA was then extracted from dissected hippocampi following standard Qiazol protocol. Initially, tissue was manually homogenised using Dounce homogeniser. The extracted RNA was further purified on miRNAeasy columns (Qiagen, DE). The quality and quantity of the extracted RNA were analysed on a NanoDrop 1000 spectrophotometer (Thermo Fisher Scientific), followed by visual inspection of the agarose gel electrophoresis images.

### LNA-Based MicroRNA Array Profiling

Two pools were prepared for each group, Unimpaired (*n* = 3 *per* pool) and Impaired (*n* = 3 *per* pool), by adding an equal amount of RNA extracted from single animals in their group. Each pool consisted of 2.5 µg of the total RNA that was run in parallel as technical replicates. For the microarray condition, we compared the Unimpaired vs the Impaired that were labelled in red (Hy5) and green (Hy3), respectively, using the Hy5/Hy3 miRCURY LNA miRNA array power labelling kit (Exiqon, DK). Fluorochrome-labelled RNA samples were then combined, denatured and hybridised to custom made slides containing LNA-modified microRNA capture probes targeting all human, mouse and rat miRNAs listed in the miRBase Sequence Database Release v.11.0 (http://microrna.sanger.ac.uk/sequences/). Slides, which contained 4800 unique mature miRNA sequences, have been kindly provided by Dr. V. Benes, from the Genomics Core Facility of the European Molecular Biology Laboratory (Heidelberg, Germany). The hybridization was performed according to the miRCURY LNA array manual using hybridization chambers (Agilent, California), for 16 h at 56 °C. After hybridization, the microarray slides were scanned using a ScanArray Lite microarray scanner (Packard Bioscience, USA). The image analysis was then carried out using GenePix® Pro 6 software (Molecular Devices, USA). The software flagged marginal and absent spots automatically, and these results in each slide were crosschecked manually to eliminate them. Data were then normalised using different endogenous controls present in the LNA-modified spotted library. The Hy3/Hy5 ratios were log_2_-transformed. Only probes with log intensity > 8 and < 14 were taken in account, to avoid non-linear effects caused by the noise floor at low intensities or by saturation at high ones [23]. Only miRNAs with fold change above 0.5 or below − 0.5 with standard deviation > 0.4 were considered.

### RT-qPCR

Results from microRNA array were confirmed by RT-qPCR miRNA quantification. Five hundred nanograms of total RNAs from each pool (i.e. Impaired and Unimpaired) were reverse transcribed using miScript II RT Kit (Qiagen, DE) with miScript Hispec buffer (specific for mature miRNA). The cDNA synthesised was diluted to 1 ng and served as the template for subsequent qPCR analysis by using miScript SYBR Green PCR Kit (Qiagen, DE) and appropriate miScript primer assays and universal primer, and run in triplicates using the Applied Biosystems 7300 Real Time PCR system, according to manufacturer’s reaction conditions.

Data were analysed using the 2^−ΔΔCt^ method [29]. The amount of each miRNA was normalised to U6 snRNA as internal reference, and the fold change in miRNA expression of Impaired animals relative to Unimpaired was measured.

### Bioinformatic Analysis

Identification of miR-153-3p predicted and validated mRNA targets was performed using miRWalk v3.0 (http://mirwalk.umm.uni-heidelberg.de/ [30]. Prediction analysis in miRWalk v3.0 was performed using three prediction algorithms: TaPmiR (binding probability was set to > 0.95), TargetScan and miRDB. Only target mRNAs with 3′-UTR binding sites predicted by at least two out of the three algorithms were selected for further analysis. Validated targets were identified by miRTarBase. STRING database (v.11.5) was used to perform the protein–protein interaction (PPI) network analysis (https://string-db.org/, [31]. STRING assumed the whole genome as statistical background for the enrichment analysis. To obtain insight into the biological function of the selected miR-153-3p gene targets, we performed KEGG pathway analysis and Geneset enrichment analysis using ShinyGO 0.77 (http://bioinformatics.sdstate.edu/go/, [32], with the size of annotated gene sets for testing limited to 5–700 genes as lower/upper limit.

### Stereotaxic Surgery

Starting from the day before surgery and until the day after, animals’ cages were provided with paracetamol (Tachipirina, Angelini, Italy) diluted in water to obtain a final concentration of 2 mg/ml and administered in drinking water. Mice were individually placed in the induction chamber, and anaesthesia was induced with 3–5% isoflurane (Iso-Vet, Piramal Healthcare UK) until loss of reflexes was achieved. Once asleep, mice were mounted on the stereotaxic instrument (David Kopf instruments, USA) with the help of front teeth incisor bar and lateral zygomatic cups. A nose mask connected to the anaesthetic tank (Harvard Apparatus) supplied continuous 2% isoflurane to maintain anaesthesia until surgery was completed. Animal’s eyes were protected with Lacrigel eye gel (Bracco, Italy). The scalp was disinfected with Betadine solution (10%, Medapharma, Italy), and a small incision was made to expose the calvarium. Briefly, to deliver the LNA or scramble solution in the dorsal hippocampus, the cranium has been pierced with a microdrill to allocate two iron cannulas (Unimed SA, Switzerland, length = 7 mm; diameter = 0.5 mm) at the following coordinates according to the mouse brain atlas (Franklin and Paxinos, 1997): AP, − 1.8; ML, ± 1.5; and DV, − 1.2. Guide cannulae were bilaterally fixed to the skull with dental acrylic (Riccardo Ilic, Italy). After surgery, animals were left undisturbed until the complete awakening and then placed back in the animal house. Mice were allowed to recover from surgery in their home cages for a week.

#### In vivo Focal Injection of Mimic or Scramble miRNA Solutions

One day after the surgery, mice were handled for 3 min for 2 days. On the day of injection, mice were gently restrained for injection needle (Unimed SA, Switzerland, length = 7.5 mm; diameter = 0.12 mm) insertion into one guide cannula at a time. The injection needle was connected through a plastic tube (internal diameter 0.86 mm, external diameter 1.52 mm, PE 20, Micromedical Tubing, 2Biological Instruments SNC, Italy) with a 1 µl Hamilton syringe (Scientific Glass Engineering, Australia). Then, 200 µM of saline-formulated (0.15 M NaCl) LNA preparations mimic miR-153-3p (Exiqon, mimic miR-153-3p, product no: 470930–001) or mimic negative control (scramble; Exiqon, product no: 479903–001) were delivered into the dorsal hippocampus at a rate of 0.4 μl *per* 2 min, using a micropump (Harvard Apparatus, Holliston, MA, USA). The needle was left in place for an additional 2 min to allow diffusion of the preparation. The injection was then repeated on the other side of the brain using the same procedure. During the injections, mice were awake and free to move in the holding cage. Behavioural testing took place 3 h after the injection procedure.

### Histology

To verify for the placement of injection sites, animals were immediately sacrificed by isoflurane anaesthesia after behavioural testing. Brains were dissected out, fixed with 4% formaldehyde solution and stored at 4 °C for at least 3 days. Ninety-micrometre thick coronal brain sections were cut with a freezing microtome. Sections were stained with cresyl violet, and injection sites were determined by examining them with a stereomicroscope (Zeiss). Only animals with a correct injector-placement were included in the statistical analysis.

### Data Collection, Analysis and Statistics

Data were analysed with the software: Statistica 8, GraphPad Prism 8.01 and G*power 3.1. For all the analyses, the level of significance was set to *p* < 0.05. Data are expressed as mean ± standard errors (SEM). The number of mice per group was calculated a priori with power analysis using G^*^Power 3.1 software with *α* = 0.05 and power (1 − *β*) = 0.80, and data were inspected for normal distribution through a Shapiro–Wilk test. Mouse behaviour in the 6-DOT was monitored and recorded during all the procedures. Object exploration was scored with the software Timer 1.3 (for Mac, NIMH, Bethesda, MD, USA). Behavioural procedures started with habituation phase; animals allowed to freely explore the empty arena were observed, and parameters such as peripheral sector crossings, central sector crossing, leaning, rearing and grooming were manually recorded offline as number of crossings or seconds spent leaning, grooming or tearing. Data collected were plotted in histograms and, when appropriate, analysed by unpaired Student’s *t* test between groups. A one-way repeated measures ANOVA for objects exploration (six levels) was performed on data obtained for the study phase and test phase of the whole 12-month group. After applying the criterion, object exploration for Unimpaired and Impaired mice of the 12 months group was analysed with a two-way repeated measures ANOVA for objects exploration (six levels) as repeated measure and behavioural performance (two levels: Unimpaired, Impaired) as between factor for both study and test phase. For the overexpression of the miR-153-3p in 3-month-old mice, object exploration was analysed with a two-way repeated measures ANOVA for objects exploration (six levels) as repeated measure and treatment (two levels: mimic and scramble) as between factor for both the study and test phase. Post hoc multiple comparisons were carried out when allowed: Dunnett’s test was used for within group comparison of the new and the familiar objects, while the Tukey’s HSD post-hoc test was used for between group comparisons.

The percentage of *new object* exploration between groups (Unimpaired and Impaired or mimic and scramble) was analysed by unpaired Student’s *t* test.

In the microarray, experiment data from two technical replicates were averaged for each microRNA, and only probes with a log_2_-ratio above + 0.7 or below − 0.7 were considered a significant variation. Correlation between microarray analysis and RT-qPCR was analysed by Pearson’s correlation test.

## Results

### High Memory Load Reveals Subtle STM Deficit in a Subgroup of Middle-Aged Mice

**Fig. 1:**
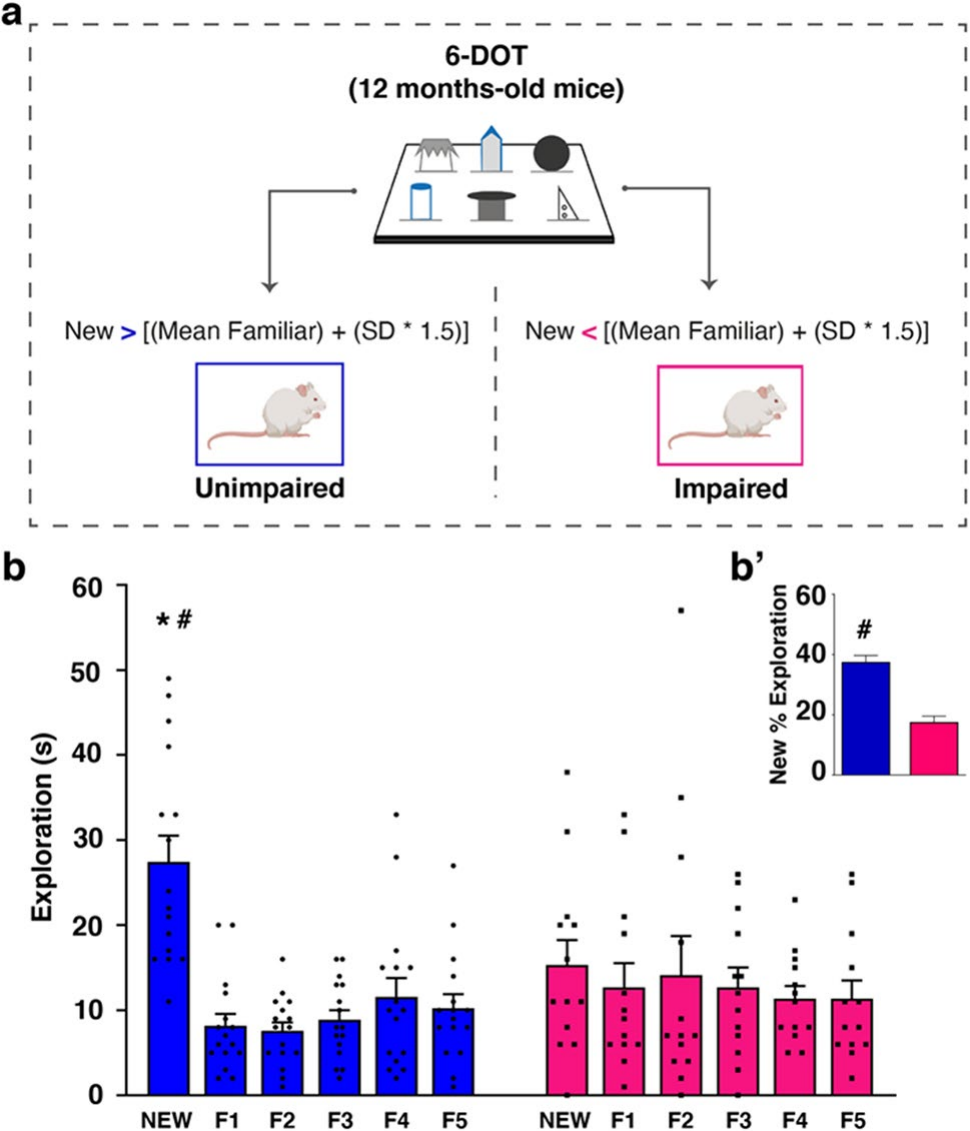
The 6-DOT reveals two groups of mice based on their high-load STM performance. **a** 12-month-old CD1 male mice underwent the 6-DOT and were segregated in two groups based on the performance. **b** After applying the criterion, mice were segregated in Unimpaired (UM; *N* = 16) and Impaired groups (IM; *N* = 13); Unimpaired mice exhibited significantly more exploration of the new object compared to Impaired mice. *p < 0.001 (Dunnett post-hoc test within group), #*p* < 0.001 (Tukey HSD post-hoc test between groups). **b′** Insert shows the difference in new object percentage of exploration between Unimpaired and Impaired mice. #*p* < 0.001 (unpaired student *t*-test)

The main aim of our work was to delineate miRNAs profile differences between Unimpaired and Impaired middle-aged mice. To this aim, we first tested middle-aged outbred mice (12-month-old CD1 males) in the 6-DOT, which allows to identify subtle HP-dependent memory deficits with prognostic value on worsening performance at later age stages [9]. Thus, based on their performance in the 6-DOT, mice were a posteriori divided in two subgroups (see methods for criterion): *Unimpaired mice* (UM), able to discriminate the new object among six different, and *Impaired mice* (IM), unable to successfully perform the task (Fig. 1a). From an initial population of 29 mice, we identified 16 subjects that fell into the UM group (55.2% of the total population) and 13 subjects that fell into the IM group (44.8%), resembling the distribution obtained in a previous study with mice of the same age and genetic background [9]. Although the overall performance of mice was good when considering the whole population (Fig. S1a-b, one-way repeated measures ANOVA for objects at test phase (Fig. S1a): *F*_(5,140)_ = 8.863, *p* < 0.0001; one-way repeated measures ANOVA for objects at study phase (Fig. S1b): *F*_(5,130)_ = 2.772, *p* = 0.0206), when comparing the two groups, we found a significant interaction between the factors “objects” and “performance” (Fig. 1b, two-way repeated measures ANOVA, effect for: objects *F*_(5,135)_ = 8.724, *p* < 0.0001; performance *F*_(1,27)_ = 0.096, *p* = 0.7596; object*performance F_(5,135)_ = 5.297, *p* = 0.0002) and a significant difference in the percentage of new object exploration (Fig. 1b′, unpaired student *t*-test: df = 27, *p* < 0.0001). The differences observed were not due to exploration bias during the study phase, as mice from the two groups explored the objects similarly in this phase (Fig. S1c; two-way repeated measures ANOVA, effect for: object *F*_(5,125)_ = 2.819, *p* = 0.0190; performance *F*_(1,25)_ = 0.144, *p* = 0.7076; object*performance *F*_(5,125)_ = 0.888, *p* = 0.4912), and they showed an overall similar amount of total exploration (Fig. S1d; unpaired student *t*-test: df = 25, *p* = 0.7076). This result confirms previous evidence showing that when challenged with a large number of items to be remembered, a fair proportion of middle-aged subjects shows an early deficit in STM ability [9].

### Unimpaired and Impaired Middle-Aged Mice Show a Different Profile of miRNA in the HP

**Fig. 2:**
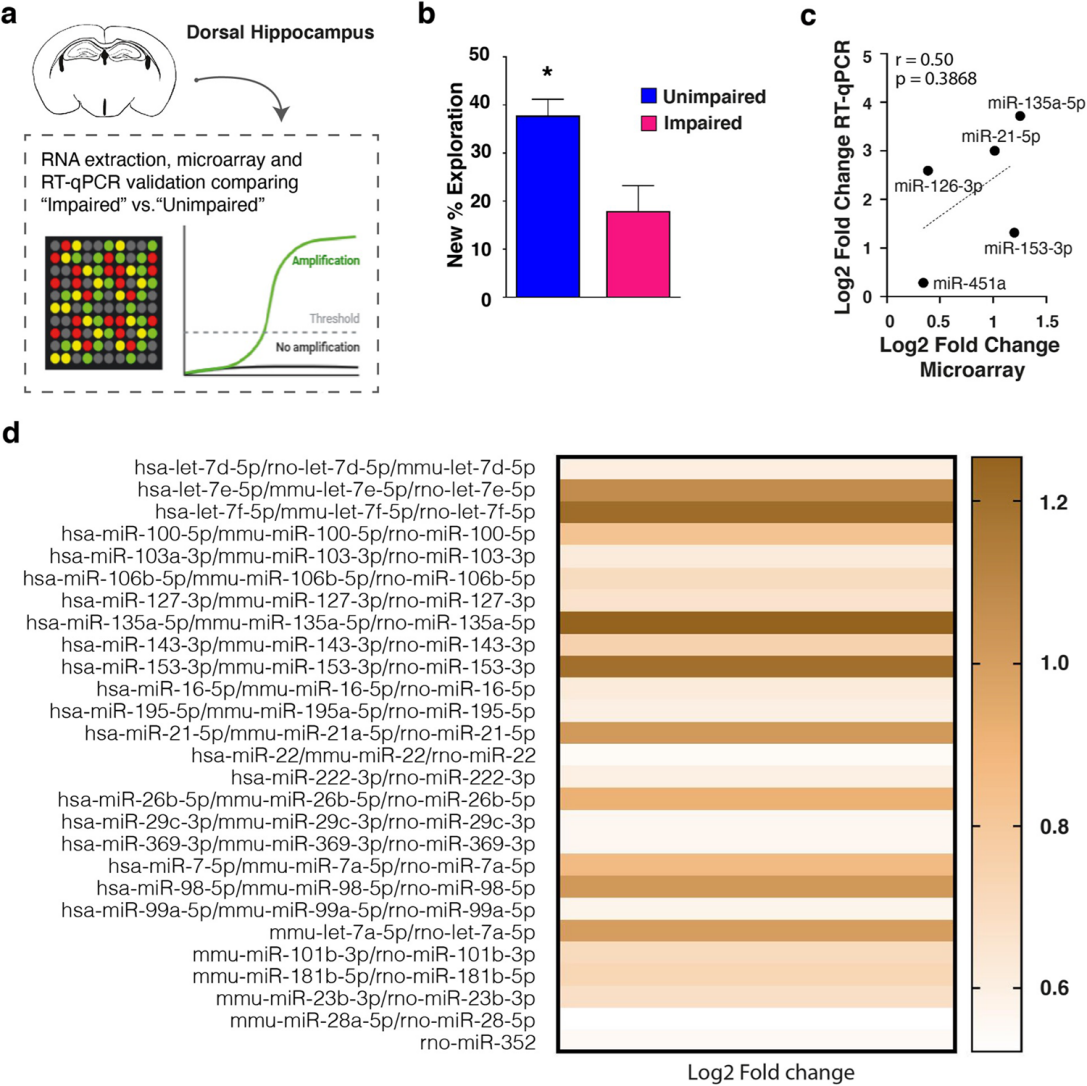
miRNA HP profile differs between Unimpaired and Impaired mice. **a** Unimpaired and Impaired hippocampi were dissected and pooled for RNA extraction and LNA-based microRNA array profiling. **b** Bar charts represent new object percentage of exploration for Unimpaired and Impaired mice selected for RNA extraction (Unimpaired vs Impaired animals, 3 *per* pool). *p < 0.05 between groups (unpaired student *t*-test). **c** Pearson’s correlation between the Unimpaired/Impaired log2 fold change from RT-qPCR and microarray analysis. **d** The heat map shows miRNAs with a log2 fold change higher than + 0.5 between Unimpaired and Impaired mice

As above-mentioned, ageing is a condition that alters miRNA expression [16–18]; however, to our knowledge, less is known about their involvement in the early stages of ageing, when only some individuals show subtle memory deficits similar to the one we showed above (Fig. 1b–b′). In the attempt to define a miRNA profile associable with MCI, two pools of representative individuals from UM (*N* = 3) and IM (*N* = 3) were selected and sacrificed for dHP dissection and RNA extraction, in order to perform a microarray analysis for the detection of miRNAs differentially expressed between the two groups (Fig. 2a). The comparison of the percentage of new object exploration between these subgroups of UM and IM mice was significantly different (Fig. 2b, unpaired student *t*-test: df = 4, *p* = 0.0050), paralleling the results of the whole group. The microarray analysis resulted in 179 miRNAs, most of which were unchanged between groups (141 miRNAs), none was downregulated, and 38 were upregulated in the IM group. To validate these results, we analysed some of the varied and unvaried miRNAs by RT-qPCR (Table 1), observing a moderate positive correlation between the IM/UM log2 fold change obtained from the RT-qPCR and the microarray analyses (Fig. 2c, Pearson’s correlation: *r* = 0.50, *p* = 0.3868). Among the miRNAs obtained, we show in Fig. 2d those whose variation in IM reached at least a + 0.5 log2 fold change compared to UM.

**Table 1:** The table shows the mean fold change (2^–∆∆Ct^ + upper |− lower error) *per* pool of UM (*N* = 3) and IM (*N* = 3) mice for the miRNAs analysed by RT-qPCR

miRNA	Pool	2^–∆∆Ct^	Upper error	Lower error
mir-135a-5p	UM	1.0000	0.358	0.263
	IM	13.1587	1.453	1.309
mir-451	UM	1.0000	0.135	0.119
	IM	1.2166	0.053	0.051
hs-miR-126	UM	1.0000	0.180	0.153
	IM	6.0362	3.414	2.181
hs-miR-21-5p	UM	1.0000	0.200	0.166
	IM	8.0140	3.008	2.187
mir-153-3p	UM	1.0000	0.275	0.216
	IM	2.4902	1.217	0.817

The detected upregulation of these miRNAs in IM suggests their potential role in early memory deficits, meriting further investigation. Among the miRNAs with the highest upregulation (fold change > 1.0) in IM, as revealed by the microarray analysis, miR-153-3p stood out due to its validated and putative targets. Indeed, we analysed miR-153-3p targets with miRWalk v3.0, identifying 101 target genes, experimentally validated or predicted by at least two out of the three algorithms used by miRWalk (Table S1). Many of these genes were of interest for their implication in memory-related plasticity mechanisms or for their role in ageing processes. Consequently, we further investigated the possible mechanisms impacted by its upregulation. The PPI network analysis conducted with STRING [31] showed a significant association among the 101 targets of miR-153-3p (PPI enrichment *p*-value = 9.89 × 10^−5^), indicating that the proteins have more interactions among themselves (71 edges, from known and predicted interactions) than what would be expected for a random set of proteins of similar size, drawn from the genome (expected number of edges, 44), with an average node degree of 1.41 and maximum degree of 12 (Fig. S2). This analysis suggests that the miR-153-3p mRNA targets are at least partially biologically connected and highlighted some high-degree nodes that could exert a pivotal role in age-related cognitive decline, such as Pten (degree = 12), Pik3r1 (degree = 10) and Adam10 (degree = 8). Next, we focused on the possible functional role of miR-153-3p, using the list of 101 targets as input for a KEGG pathways and Gene Ontology (GO) analysis. We identified several significantly enriched pathways relevant for memory-related synaptic plasticity. The KEGG pathway analysis (Table S2) revealed that miR-153-3p targets are significantly associated with many signalling pathways, such as ErbB, Sphingolipid, Phosphatidylinositol, Wnt, MTOR, cGMP-PKG, MAPK and PI3K-Akt. Interestingly, we also found a significant enrichment in pathways related to ageing (Alzheimer disease, autophagy) and neuron morphology (axon guidance, focal adhesion, regulation of actin cytoskeleton). The GO analysis revealed a strong enrichment for terms related to neuron morphology in the “Biological Process” category (Table S3). In the “Cellular component” group, we found many significant terms related to the synapse (Table S4). In the “Molecular function” category, miR-153-3p targets were significantly enriched for signalling pathways (Insulin, Ubiquitin, MAPK, Wnt, Serine/threonine phosphatase), as shown in Table S5.

These analyses strongly suggest a possible role of miR-153-3p and its targets in the STM deficit observed in IM.

### Overexpression of miR-153-3p is Sufficient to Cause an Impairment in High-Load STM in Adult Mice

**Fig. 3:**
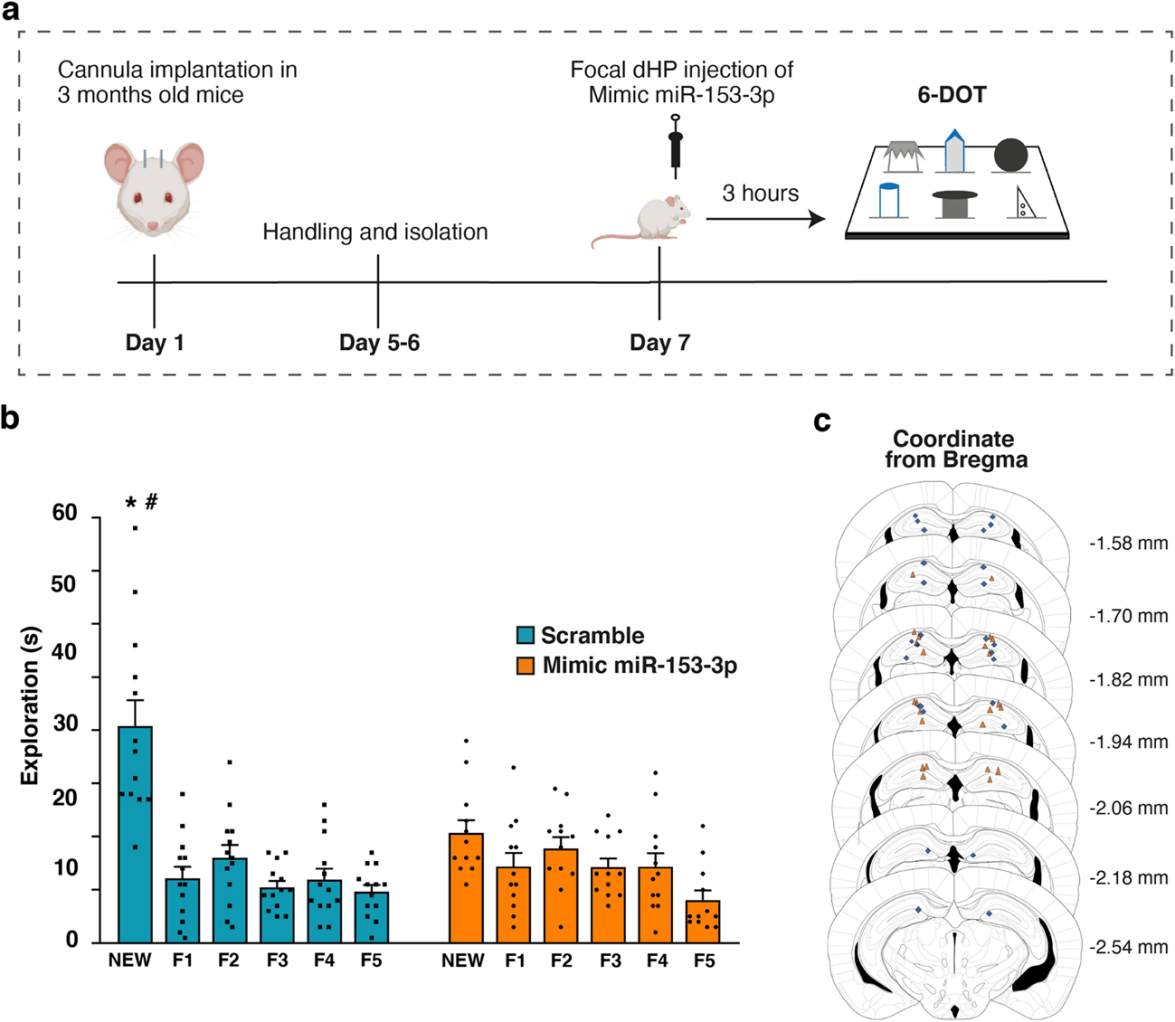
Upregulation of the miR-153-3p in the HP impairs performance in the 6-DOT in adult mice. **a** After surgery and handling, 3-month-old mice were injected with mimic-miR-153-3p 3 h prior to performing the 6-DOT. **b** Bar charts show objects exploration for controls (scramble) and mimic-miR-153-3p injected mice. Only control mice (*N* = 13) were able to discriminate the new object compared to all the other familiar ones (F1-F5), while mimic-miR-153-3p injected mice (*N* = 12) showed no difference in exploration between the objects at test and a significantly lower exploration of the new object compared to controls. *p < 0.001 (Dunnett post-hoc test within group), #*p* < 0.001(Tukey HSD post-hoc test between groups). **c** Illustration of the placement injections in the dHP of control (cyan) and mimic-miR-153-3p injected (orange) mice

Although the detected upregulation of miR-153-3p in Impaired mice suggests its possible implication in the STM impairment, to demonstrate whether it had a causal role, we upregulated miR-153-3p in the HP by specific miRNA mimic local injection in a group of adult mice. Thus, 3-month-old CD1 male mice were implanted with cannulae above the HP in order to allow in vivo infusion of exogenous mimic miR-153-3p (*N* = 12) or scramble negative control (*N* = 13) 3 h before the execution of 6-DOT (Fig. 3a).

We found that miR-153-3p overexpression in adult mice was able to mimic the cognitive impairment observed in the middle-aged IM, as mimic injected animals were not able to discriminate the new object among the familiar ones in respect to scramble injected mice (Fig. 3b, two-way repeated measures ANOVA for object *F*_(5,115)_ = 23.056, *p* < 0.0001; treatment *F*_(1,23)_ = 1.047, *p* = 0.3169; object*treatment *F*_(5,115)_ = 8.008, *p* < 0.0001). Also in this latter experiment, impaired novel object discrimination during the test phase in the experimental group was not due to a specific effect of the treatment on exploratory behaviour, as no differences between groups were observed on locomotor activity and vertical exploration (Fig. S3a-e, unpaired student *t*-test: peripheral sector crossing (Fig. S3a) df = 23, *p* = 0.3136; central sector crossing (Fig. S3b) df = 23, *p* = 0.0775; leaning (Fig. S3c) df = 23, *p* = 0.9918; rearing (Fig. S3d) df = 23, *p* = 0.3892; grooming (Fig. S3e) df = 23, *p* = 0.1900). Neither single object exploration during the study phase nor total exploration showed differences between the two groups (Fig. S3f-g; two-way repeated measures ANOVA for single object exploration (Fig. S3f) effect for object *F*_(5,115)_ = 9.475, *p* < 0.0001, treatment *F*_(1,23)_ = 1.798, *p* = 0.1930, object*treatment *F*_(5,115)_ = 0.617, *p* = 0.6874; unpaired student *t*-test: total exploration during study phase (Fig. S3g) df = 23, *p* = 0.1930). Overall, these results suggest that the upregulation of the miR-153-3p leads to STM impairment in the 6-DOT.

## Discussion

In the present study, we identified for the first time a dysregulation of miRNA expression profile in the HP of middle-aged mice showing a STM impairment (IM), as compared to Unimpaired mice of the same age (UM), and we demonstrated that upregulation of miR-153-3p expression is causally involved in this type of deficit.

It has recently been demonstrated that middle-aged male mice which are impaired in the 6-DOT show altered hippocampal post-transcriptional regulation of GluA1, characterised by lower GluA1 receptor phosphorylation at the synaptosomes, as well as deficits of autophagy, involving the blockade of the late stages of the autophagic flux, associated with higher amyloid-beta and alpha-synuclein proteins aggregation [9]. Our findings on altered miRNA profile in IM compared with UM further extend this body of evidence and suggest that the impairment of STM may begin at the level of miRNA expression. Indeed, some miRNAs that were highly upregulated in the HP of IM are already known for their involvement in memory-related plasticity mechanisms. In particular, miR-135a-5p, the most upregulated miRNA in IM, has a well-established role in the presynaptic modulation of excitatory neurotransmission [23, 25] and in AD-related memory and synaptic disorders [33].

MiR-153-3p ranked third among the differentially expressed miRNAs in the microarray. It attracted our attention as a potential new candidate in regulating key mechanisms related to memory during early ageing, due to its validated and predicted targets. Indeed, the PPI network analysis conducted with STRING indicated that many miR-153-3p targets are functionally connected and enriched for proteins involved in synaptic plasticity and age-related cognitive decline. Accordingly, here we showed that injection of mimic-miR-153-3p in the HP of young mice—3 h before the behavioural procedure—induces a deficit in the 6-DOT, demonstrating that the upregulation of miR-153-3p in the HP is sufficient to induce an impairment of STM, without affecting the exploratory and locomotor activity of mice. This result corroborates the importance of a functionality intact HP when coping with high memory load [8, 9, 14, 16, 34]. Furthermore, it suggests that the observed increase in miR-153-3p miRNA expression in the HP of middle-aged IM was not only directly related to their cognitive performance, but also occurred before and independently of the learning experience itself, rather than as a result of it. It could be speculated that the upregulation of miR-153-3p, whether occurring during ageing or induced artificially in younger mice, sets the stage for STM impairment.

MiR-153-3p has been shown to disrupt the interaction between Beclin-1 and the B-cell lymphoma-2 gene (Bcl-2) by downregulating Bcl-2 [35], an interaction that physiologically facilitates autophagy, whose blockade was previously demonstrated to be functionally involved in the STM deficits of IM [9]. Moreover, this miRNA has been shown to regulate α-synuclein at the post-transcriptional level—by binding to the 3′-untranslated region of α-synuclein and down-regulating its mRNA and protein levels [36]—and it is intriguing that dysfunctional α-synuclein phosphorylation was previously shown to occur in IM [9]. Finally, miR-153-3p is also known to participate in neuroprotective mechanisms [37], which might lead to age-dependent memory deficits as a secondary effect. Indeed, increased levels of miR153-3p were found in the HP of a rat model of chronic brain hypoperfusion [38]—a condition often associated with age-related disease, such as Alzheimer’s disease (AD) and vascular dementia [39, 40]—and it has been demonstrated that this upregulation was causally related to decreased pre- and post-synaptic plasticity at the CA3-CA1 pathway, reduced expression of several vesicle fusion proteins and presynaptic proteins (SNAP-25, VAMP-2, syntaxin-1A and synaptotagmin-1, synapsin I) and altered synaptic vesicle trafficking [34, 38].

MiR-153-3p upregulation could also have an indirect effect on STM through the dysregulation of calcium signalling cascades that have a well-known role in synaptic plasticity and cognitive functions [41, 42]. Indeed, both the cytosolic calcium levels and its release from intracellular stores could be regulated by miR-153-3p, acting on calcium release from the ER [43] or interfering with Cacna1c expression [44]. There is evidence that in the advanced stage of AD, miR-153-3p is downregulated and Cacna1c is increased. Higher than normal levels of Cacna1c were observed in the HP of 6-month-old double transgenic mice APPswe/PS1ΔE9 (APP/PS1) when their cognitive abilities (spatial learning and memory) were already compromised [45]. In addition, Long et al. [46] observed that miR-153-3p is reduced in a subgroup of AD patients at an advanced stage of the disease and they also demonstrated that administration of miR-153-3p to human foetal brain cultures reduced the expression of amyloid-beta precursor protein (APP) by targeting its mRNA 3′-untranslated region (3′-UTR) [46, 47]. Although these data seem at odds with ours, it should be noted that the stages of cognitive impairment and disease progression in these studies are different than in our mouse population. The brain samples of AD patients in the study by Long et al. (2012) were from individuals at an advanced stage of AD progression (Braak stages III/IV), when cognitive decline is generally already defined as mild/moderate [48], whereas mice in the study by Jiang et al. [45] were already impaired in several memory tasks. Our IM group is a model of MCI [9], not AD, and to our knowledge, there is no characterisation of miR-153-3p in patients with MCI, leaving open the hypothesis that overexpression of this miRNA may represent an attempt to counteract the increasing levels of amyloid-beta and alpha-synuclein production at this stage.

A limitation of our study is the lack of specific analyses of changes in the expression of miR-153-3p targets. Future studies should investigate specific miR-153-3p regulated pathways and mechanisms that become dysfunctional in middle-aged Impaired mice.

Our data suggest that miRNA profiling and manipulation could be an important adjunct respectively in early diagnosis and prevention of age-related cognitive decline. In conclusion, although further studies are needed to identify the biological significance of the miR-153-3p upregulation that we observed in early STM-Impaired mice, the identification of a causal relationship between the miR-153-3p levels and STM performance that we have shown here in young mice highlights this miRNA as a potential candidate as a marker of early cognitive decline and for future efforts to prevent early cognitive deficits in ageing.

### Supplementary Information

Below is the link to the electronic supplementary material.

Supplementary file1 (DOCX 1.01 MB)

## Data Availability

All data supporting described findings can be obtained from the corresponding authors (Andrea Mele and Arianna Rinaldi) upon reasonable request.

## Abbreviations

6-DOT: 6 Different object task
AD: Alzheimer’s disease
ADAM23: Disintegrin and metalloproteinase domain-containing protein 23
AMPA: α-Amino-3-hydroxy-5-methyl-4-isoxazole propionic acid
APP/PS: APPswe/PS1ΔE9
APP: Amyloid-beta precursor protein
Bcl-2: B-cell lymphoma-2 gene
cDNA: Complementary deoxyribonucleic acid
dHP: Dorsal hippocampus
ER: Endoplasmic reticulum
HP: Hippocampus
HSD: Honestly significant difference
IM: Impaired mice
KEGG: Kyoto encyclopaedia of gene and genome
LNA: Locked nucleic acid
MCI: Mild cognitive impairment
miRNA: MicroRNA
mRNA: Messenger ribonucleic acid
Pcdh8: Protocadherin-8
PCR: Polymerase chain reaction
PE: Polyethylene
PPI: Protein-protein interaction
PSC: Peripheral sector crossing
RNA: Ribonucleic acid
RT-qPCR: Reverse transcription-quantitative PCR
SD: Standard deviation
SEM: Standard error of the mean
STM: Short-term memory
UM: Unimpaired mice
