# Supplementary material for: Short-Term Memory Deficit Associates with miR-153-3p Upregulation in the Hippocampus of Middle-Aged Mice
Source: Mol Neurobiol. 2023 Nov 15;61(5):3031–41. doi: 10.1007/s12035-023-03770-5 (PMC11041712; doi:10.1007/s12035-023-03770-5)
Supplement: Supplementary file 1 — Supplementary file1 (DOCX 1.01 MB) [file 12035_2023_3770_MOESM1_ESM.docx]

**Supplementary Information**

**Short-term memory deficit associates with miR-153-3p upregulation in the hippocampus of middle-aged mice**

**Authors**

F. Stabile^1,2^#, G. Torromino^1,3^#, S. Rajendran^1,2^, G. Del Vecchio^1^, C. Presutti^1^, C. Mannironi^4^, E. De Leonibus^5,6^, A. Mele^1,2^ *, A. Rinaldi^1,2^ **

**Affiliations**

^1^ Department of Biology and Biotechnologies 'Charles Darwin' (BBCD), Sapienza University of Rome, Italy.

^2^ Centre for Research in Neurobiology Daniel Bovet (CRiN), Sapienza University of Rome, Italy.

^3^ Department of Humanistic Studies, University of Naples Federico II, Italy.

^4^ Institute of Molecular Biology and Pathology, National Research Council, c/o Department of Biology and Biotechnology, Sapienza University of Rome, Italy.

^5^ Institute of Biochemistry and Cell Biology, National Research Council (IBBC-CNR), Monterotondo (Rome), Italy.

^6^ Telethon Institute of Genetics and Medicine (TIGEM), Pozzuoli (Naples), Italy.

** arianna.rinaldi@uniroma1.it

* andrea.mele@uniroma1.it

# These authors contributed equally

**
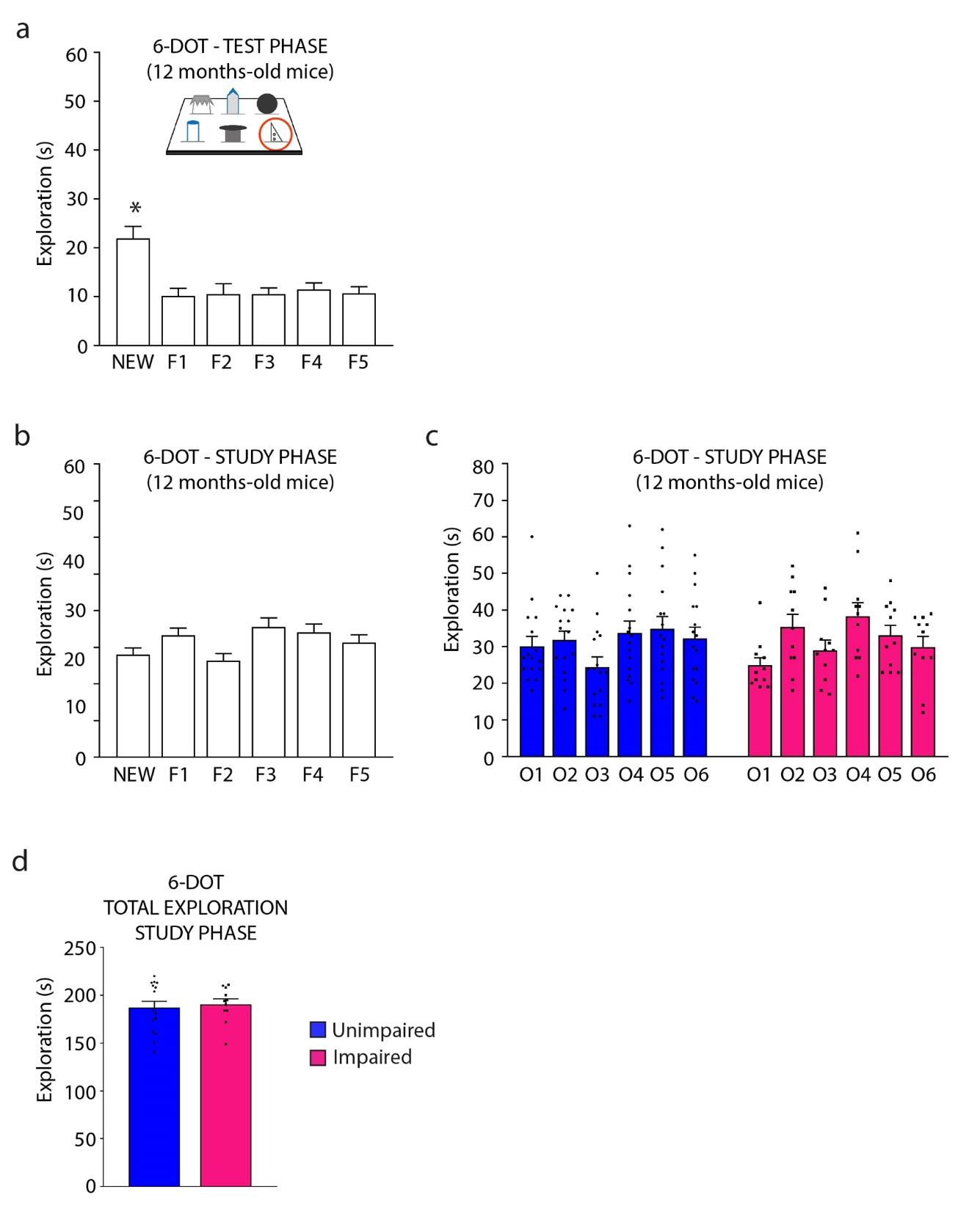
**

**Fig. S1**

**a.** Bar charts represent object exploration of 12 months old mice (N = 29) during the test phase of the 6-DOT. New object was significantly more explored compared to all familiar objects. * p < 0.001 (Dunnett post-hoc test).

**b.** Bar charts represent object exploration of 12 months old mice (N = 29) at the 6-DOT study phase. All the objects were similarly explored during the study phase.

**c.** Bar charts show no difference in single objects exploration of 12 months old mice during the study phase; mice were segregated based on their performance at the test in Unimpaired (blue; N = 16) and Impaired (magenta; N = 13).

**d.** Total exploration during the study phase of Unimpaired and Impaired groups was similar between groups (N_UM_ = 16; N_IM_ = 13).


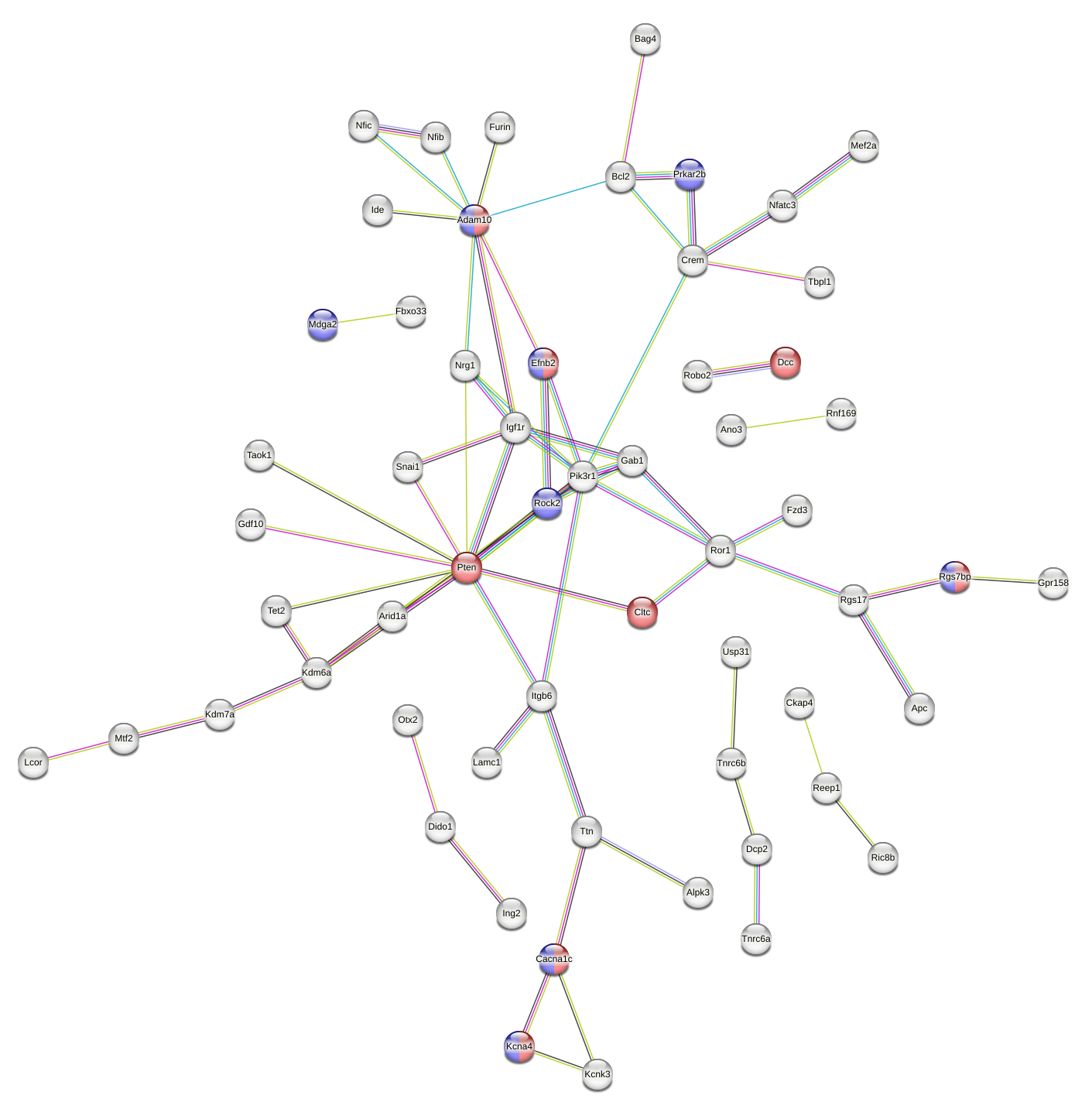


**Fig. S2**

Protein-protein association network for miR-153-3p targets. The network nodes represent individual proteins. Colored nodes highlight proteins in the GO cellular component term “synaptic membrane” (red) and “glutamatergic synapse” (blue). The network edges represent protein-protein associations, known from curated databases (light blue), experimentally determined (pink), or predicted from gene co-expression (black), co-occurence (blu), or from textmining (yellow). Only connected nodes are shown in the network.

**
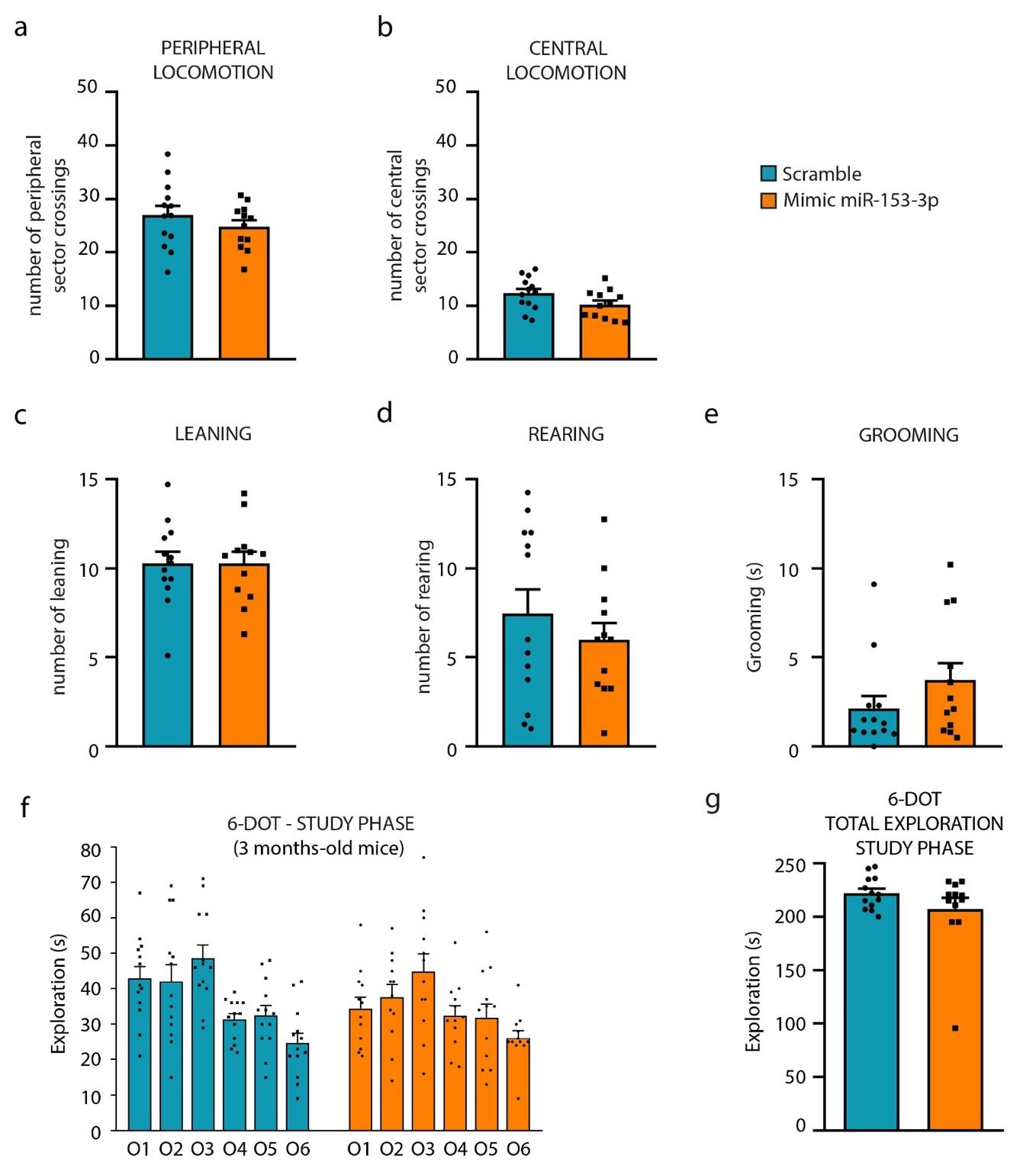
**

**Fig. S3.**

**a-b.** No significant differences were observed in locomotor activity between scramble (N = 13) and mimic-miR-153-3p (N = 12) injected groups for peripheral **(a)** or central **(b)** locomotion.

**c-e.** No significant differences between scramble and mimic-miR-153-3p injected mice were observed in exploratory activity in the number of leaning **(c)** or rearing **(d)**, neither in the seconds spent in grooming behaviour **(e)**.

**f-g.** Single objects exploration at the study phase did not significantly differ between scramble and mimic-miR-153-3p injected mice **(f)**, and the total exploration time at the study **(g)** phases was also similar.

**Table S1**

List of miR-153-3p targets predicted and validated by at least two of the three prediction algorithms (TarPmiRNA – binding probability set to >0.95 –, TargetScan and miRDB) in miRWalk v3.0. Validated genes are highlighted in blue.


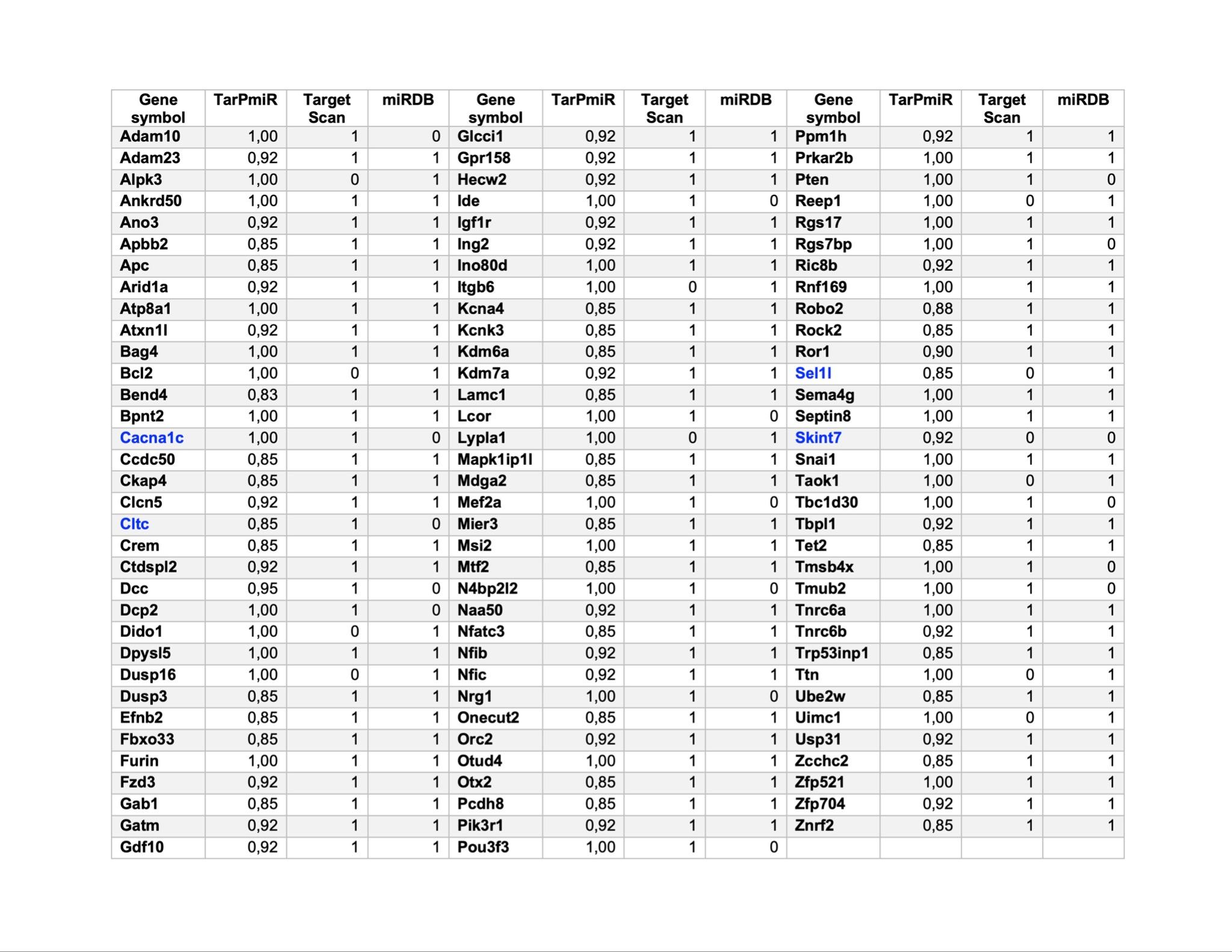


**Table S2**

KEGG pathway analysis of miR-153-3p target genes. FDR < 0.05 was considered significant.

| **Pathway** | **Pathway Genes** | **N of Genes** | **Fold Enrich.** | **FDR** | **Genes** |
| --- | --- | --- | --- | --- | --- |
| EGFR tyrosine kinase inhibitor resistance | 79 | 6 | 16.5 | 0.00015 | Igf1r Pten Gab1 Pik3r1 Bcl2 Nrg1 |
| Axon guidance | 181 | 9 | 10.8 | 0.00003 | Efnb2 Fzd3 Rock2 Sema4g Dpysl5 Nfatc3 Pik3r1 Robo2 Dcc |
| Cortisol synthesis and secretion | 72 | 3 | 9.0 | 0.03257 | Kcna4 Kcnk3 Cacna1c |
| ErbB signaling pathway | 84 | 3 | 7.7 | 0.04286 | Gab1 Pik3r1 Nrg1 |
| Focal adhesion | 200 | 7 | 7.6 | 0.00173 | Igf1r Pten Rock2 Lamc1 Itgb6 Pik3r1 Bcl2 |
| Sphingolipid signaling pathway | 122 | 4 | 7.1 | 0.02253 | Pten Rock2 Pik3r1 Bcl2 |
| Phosphatidylinositol signaling system | 96 | 3 | 6.8 | 0.04941 | Pten Pik3r1 Bpnt2 |
| MicroRNAs in cancer | 162 | 5 | 6.7 | 0.01347 | Apc Fzd3 Pten Pik3r1 Bcl2 |
| Wnt signaling pathway | 167 | 5 | 6.5 | 0.01359 | Apc Fzd3 Rock2 Nfatc3 Ror1 |
| Autophagy | 140 | 4 | 6.2 | 0.03189 | Igf1r Pten Pik3r1 Bcl2 |
| Signaling pathways regulating pluripotency of stem cells | 140 | 4 | 6.2 | 0.03189 | Igf1r Apc Fzd3 Pik3r1 |
| MTOR signaling pathway | 156 | 4 | 5.6 | 0.03952 | Igf1r Fzd3 Pten Pik3r1 |
| CGMP-PKG signaling pathway | 171 | 4 | 5.1 | 0.04672 | Rock2 Mef2a Nfatc3 Cacna1c |
| Regulation of actin cytoskeleton | 219 | 5 | 4.9 | 0.03018 | Apc Rock2 Itgb6 Pik3r1 Tmsb4x |
| MAPK signaling pathway | 294 | 6 | 4.4 | 0.02253 | Dusp3 Igf1r Taok1 Dusp16 Nfatc3 Cacna1c |
| PI3K-Akt signaling pathway | 357 | 6 | 3.6 | 0.04008 | Igf1r Pten Lamc1 Itgb6 Pik3r1 Bcl2 |
| Alzheimer disease | 380 | 6 | 3.4 | 0.04672 | Apc Fzd3 Pik3r1 Cacna1c Adam10 Ide |

**Table S3**

Analysis of enriched gene ontology (GO) terms in the “Biological process” category for miR-153-3p target genes. Redundant terms and cancer-specific terms were not included in the table. FDR < 0.05 was considered significant.

| **Biological Process** | **Pathway Genes** | **N of Genes** | **Fold Enrich.** | **FDR** | **Genes** |
| --- | --- | --- | --- | --- | --- |
| Reg. of synaptic membrane adhesion | 6 | 2 | 72.2 | 0.0284 | Mdga2 Pcdh8 |
| Cell-substrate junction disassembly | 6 | 2 | 72.2 | 0.0284 | Dusp3 Pik3r1 |
| Pos. reg. of hematopoietic stem cell proliferation | 6 | 2 | 72.2 | 0.0284 | N4bp2l2 Atxn1l |
| Histone H3-K27 demethylation | 6 | 2 | 72.2 | 0.0284 | Kdm6a Kdm7a |
| Pos. reg. of histone H3-K27 methylation | 7 | 2 | 61.9 | 0.0340 | Lcor Mtf2 |
| Reg. of transforming growth factor beta activation | 9 | 2 | 48.2 | 0.0463 | Itgb6 Furin |
| Central nervous system neuron axonogenesis | 39 | 3 | 16.7 | 0.0463 | Nfib Pten Dcc |
| Demethylation | 68 | 4 | 12.7 | 0.0284 | Otud4 Kdm6a Tet2 Kdm7a |
| Cell-substrate junction organization | 95 | 5 | 11.4 | 0.0115 | Dusp3 Pten Lamc1 Pik3r1 Bcl2 |
| Reg. of cell-matrix adhesion | 119 | 5 | 9.1 | 0.0284 | Dusp3 Pten Pik3r1 Onecut2 Bcl2 |
| Axon guidance | 235 | 9 | 8.3 | 0.0005 | Efnb2 Fzd3 Nfib Otx2 Sema4g Dpysl5 Apbb2 Robo2 Dcc |
| Reg. of cell junction assembly | 212 | 6 | 6.1 | 0.0340 | Dusp3 Pten Rock2 Mdga2 Pik3r1 Snai1 |
| Cell-matrix adhesion | 213 | 6 | 6.1 | 0.0340 | Dusp3 Pten Itgb6 Pik3r1 Onecut2 Bcl2 |
| Axonogenesis | 445 | 11 | 5.4 | 0.0012 | Efnb2 Fzd3 Nfib Pten Otx2 Sema4g Dpysl5 Apbb2 Robo2 Bcl2 Dcc |
| Pos. reg. of cell migration | 549 | 13 | 5.1 | 0.0005 | Apc Rock2 Sema4g Furin Gab1 Bag4 Atp8a1 Pik3r1 Snai1 Onecut2 Tmsb4x Adam10 Bcl2 |
| Cell junction assembly | 430 | 10 | 5.0 | 0.0047 | Efnb2 Dusp3 Pten Rock2 Lamc1 Mdga2 Pik3r1 Snai1 Bcl2 Nrg1 |
| Cell morphogenesis involved in neuron differentiation | 603 | 14 | 5.0 | 0.0004 | Efnb2 Fzd3 Nfib Pten Otx2 Sema4g Dpysl5 Apbb2 Mef2a Hecw2 Robo2 Adam10 Bcl2 Dcc |
| Protein dephosphorylation | 306 | 7 | 5.0 | 0.0387 | Dusp3 Pten Rock2 Dusp16 Ctdspl2 Ppm1h Bcl2 |
| Neuron projection morphogenesis | 656 | 15 | 5.0 | 0.0004 | Efnb2 Fzd3 Nfib Pten Taok1 Otx2 Sema4g Dpysl5 Apbb2 Mef2a Hecw2 Robo2 Adam10 Bcl2 Dcc |
| Axon development | 488 | 11 | 4.9 | 0.0026 | Efnb2 Fzd3 Nfib Pten Otx2 Sema4g Dpysl5 Apbb2 Robo2 Bcl2 Dcc |
| Chemotaxis | 596 | 13 | 4.7 | 0.0008 | Efnb2 Dusp3 Fzd3 Nfib Otx2 Sema4g Dpysl5 Apbb2 Gab1 Robo2 Adam10 Dcc Nrg1 |
| Neg. reg. of locomotion | 323 | 7 | 4.7 | 0.0463 | Dusp3 Pten Sema4g Trp53inp1 Robo2 Bcl2 Nrg1 |
| Dephosphorylation | 469 | 9 | 4.2 | 0.0284 | Dusp3 Pten Rock2 Dusp16 Ctdspl2 Ppm1h Bag4 Bcl2 Bpnt2 |
| Tissue morphogenesis | 639 | 10 | 3.4 | 0.0463 | Efnb2 Arid1a Fzd3 Pten Pcdh8 Kdm6a Snai1 Ttn Bcl2 Nrg1 |

**Table S4**

Analysis of enriched gene ontology (GO) terms in the “Cellular component” category for miR-153-3p target genes. Redundant terms were not included in the table. FDR < 0.05 was considered significant.

| **Cellular component** | **Pathway Genes** | **N of Genes** | **Fold Enrich.** | **FDR** | **Genes** |
| --- | --- | --- | --- | --- | --- |
| Trans-Golgi network transport vesicle membrane | 12 | 2 | 36.1 | 0.0315 | Furin Cltc |
| RISC complex | 14 | 2 | 31.0 | 0.0331 | Dcp2 Tnrc6a |
| Dendritic shaft | 68 | 4 | 12.7 | 0.0088 | Prkar2b Rgs7bp Kcna4 Cacna1c |
| Presynaptic membrane | 186 | 8 | 9.3 | 0.0003 | Efnb2 Rgs7bp Adam23 Pcdh8 Kcna4 Cltc Cacna1c Znrf2 |
| Intrinsic component of presynaptic membrane | 120 | 5 | 9.0 | 0.0085 | Efnb2 Rgs7bp Adam23 Kcna4 Cacna1c |
| Postsynaptic density membrane | 105 | 4 | 8.3 | 0.0315 | Efnb2 Rgs7bp Cacna1c Dcc |
| Integral component of presynaptic membrane | 108 | 4 | 8.0 | 0.0324 | Efnb2 Adam23 Kcna4 Cacna1c |
| Intrinsic component of postsynaptic membrane | 171 | 6 | 7.6 | 0.0058 | Efnb2 Rgs7bp Pcdh8 Kcna4 Cacna1c Dcc |
| Integral component of postsynaptic membrane | 162 | 5 | 6.7 | 0.0243 | Efnb2 Pcdh8 Kcna4 Cacna1c Dcc |
| Intrinsic component of synaptic membrane | 232 | 7 | 6.5 | 0.0053 | Efnb2 Rgs7bp Adam23 Pcdh8 Kcna4 Cacna1c Dcc |
| Integral component of synaptic membrane | 213 | 6 | 6.1 | 0.0133 | Efnb2 Adam23 Pcdh8 Kcna4 Cacna1c Dcc |
| Postsynaptic membrane | 317 | 8 | 5.5 | 0.0053 | Efnb2 Pten Rgs7bp Pcdh8 Kcna4 Cacna1c Adam10 Dcc |
| Synaptic membrane | 447 | 11 | 5.3 | 0.0007 | Efnb2 Pten Rgs7bp Adam23 Pcdh8 Kcna4 Cltc Cacna1c Adam10 Znrf2 Dcc |
| Glutamatergic synapse | 474 | 11 | 5.0 | 0.0009 | Efnb2 Prkar2b Rgs7bp Adam23 Ppm1h Mdga2 Pcdh8 Kcna4 Cacna1c Adam10 Nrg1 |
| Presynapse | 610 | 14 | 5.0 | 0.0002 | Efnb2 Clcn5 Fzd3 Septin8 Rgs7bp Adam23 Ror1 Pcdh8 Atp8a1 Kcna4 Cltc Cacna1c Adam10 Znrf2 |

**Table S5**

Analysis of enriched gene ontology (GO) terms in the “Molecular function” category for miR-153-3p target genes. Redundant terms were not included in the table. FDR < 0.05 was considered significant.

| **Molecular function** | **Pathway Genes** | **N of Genes** | **Fold Enrich.** | **FDR** | **Genes** |
| --- | --- | --- | --- | --- | --- |
| Insulin receptor substrate binding | 10 | 2 | 43.3 | 0.033 | Igf1r Pik3r1 |
| Ubiquitin-specific protease binding | 20 | 3 | 32.5 | 0.011 | Pten Lcor Cltc |
| Platelet-derived growth factor receptor binding | 14 | 2 | 31.0 | 0.042 | Pten Pik3r1 |
| MAP kinase phosphatase activity | 15 | 2 | 28.9 | 0.042 | Dusp3 Dusp16 |
| Wnt-activated receptor activity | 15 | 2 | 28.9 | 0.042 | Fzd3 Ror1 |
| 2-oxoglutarate-dependent dioxygenase activity | 50 | 3 | 13.0 | 0.039 | Kdm6a Tet2 Kdm7a |
| Protein serine/threonine phosphatase activity | 89 | 5 | 12.2 | 0.010 | Dusp3 Pten Dusp16 Ctdspl2 Ppm1h |
| Protease binding | 148 | 6 | 8.8 | 0.010 | Pten Lcor Furin Cltc Ttn Bcl2 |
| Protein tyrosine kinase binding | 105 | 4 | 8.3 | 0.038 | Dusp3 Pten Pik3r1 Nrg1 |
| Modification-dependent protein binding | 216 | 6 | 6.0 | 0.025 | Uimc1 Mtf2 Kdm7a Ide Rnf169 Ing2 |
| Ubiquitin protein ligase binding | 307 | 7 | 4.9 | 0.025 | Prkar2b Apc Ube2w Bag4 Ccdc50 Pik3r1 Bcl2 |
| Phosphatase activity | 264 | 6 | 4.9 | 0.038 | Dusp3 Pten Dusp16 Ctdspl2 Ppm1h Bpnt2 |
